# Exploring the social and emotional impact of COVID-19 on older residents of the Greater Klang Valley, Malaysia: A qualitative study

**DOI:** 10.1371/journal.pone.0332610

**Published:** 2025-10-09

**Authors:** Mirchandani Shobha, Karen Morgan, Maw Pin Tan

**Affiliations:** 1 Division of Geriatric Medicine, Department of Medicine, Faculty of Medicine, University Malaya, Kuala Lumpur, Malaysia; 2 RCSI & UCD Malaysia Campus, Penang, Malaysia; Sefako Makgatho Health Sciences University, SOUTH AFRICA

## Abstract

**Background:**

The Coronavirus Disease 2019 (COVID-19) pandemic and associated public health measures significantly disrupted the lives of older adults. Few studies have captured the firsthand experiences of this population during such unprecedented times, emphasizing the need for qualitative insights into their lived realities. This study aimed to explore the social and emotional impact of the COVID-19 pandemic on older adults, focusing on personal experience, coping mechanisms, and adaptive responses to changes.

**Methods:**

A qualitative research design was employed, utilizing snowball sampling to recruit participants aged 60 years and above. Semi-structured interviews were conducted to collect in-depth narratives about participants’ experiences post-pandemic, between May 2023- March 2024. Data were analysed using thematic analysis to identify recurring patterns and meaningful themes.

**Results:**

A total of 19 participants (aged 60–81 years, 10 women) were interviewed. Four themes emerged from the study: 1) emotional and psychological symptoms linked to infection control; 2) feeling of loss from restrictions; 3) coping strategies in adversity; 4) information and communication. Participants relied on their resilience and life experiences to navigate fear and uncertainty during the pandemic.

**Conclusion:**

The identified themes reflect a collective experience of fear, loss, adaptation and resilience. Support systems and adaptability demonstrated by individuals underscored the capacity to cope with and overcome adversity. A good social security system post-retirement is hard to achieve in low and middle income countries. Future research should utilize fundings to focus on providing targeted support to older adults and other LMICs to develop mitigation strategies to prevent negative psychological, physical and financial effects in major disasters.

## Introduction

Coronavirus disease 2019 (COVID-19) is an illness caused by the Severe Acute Respiratory Syndrome Coronavirus 2 (SARS-CoV-2), first detected during an outbreak of respiratory illness in Wuhan City, Hubei Province, China (Centers for Disease Control and Prevention, 2020). The World Health Organization (WHO) declared the outbreak a public health emergency of international concern on 30 January 2020, and later recognized it as a pandemic on 11 March 2020 (World Health Organization, 2023). Globally, the pandemic disrupted daily life, producing widespread fear, uncertainty, and psychological distress. A systematic review of 177 studies from European countries comparing mental health before and during the course of pandemic indicated that the prevalence of mental health issues, such as depression, anxiety, and other non-specific conditions, increased among general population samples following the onset of the pandemic. This trend can be viewed as an acute reaction to a global crisis that brought about significant disruption, widespread fear, financial challenges, and grief [1]. The COVID-19 pandemic has left an indelible mark on societies worldwide. Its impact is most visible not only in healthcare systems but also in how individuals and communities navigate the social, economic, and psychological repercussions of the crisis [2].

The Coronavirus 2019 (COVID-19) pandemic had significantly changed people’s daily lives, bringing feelings of fear and anxiety due to uncertainties and unpredictability challenging their physical, social and psychological conditions [3]. Early restrictions involved closing schools and workplaces, a full lockdown of businesses, cancellation of public events, limiting movement, and implementing special protection measures for older adults. Evidence from a qualitative systematic review of studies across 32 countries in 2023, indicates that older adults were considered more vulnerable to severe disease and death from COVID-19 due to age-related physiological changes and underlying health conditions [4]. Public health authorities, responded to this threat by discouraging older adults from entering supermarkets and grocery stores, introducing senior-only shopping hours at pharmacies and other essential services [5]. COVID19 lockdown policies, therefore, potentially led to increased social isolation, which disrupted the usual routines for many daily activities. Even with adaptation strategies isolation gives rise to feelings of sadness and loneliness [6]. As a result, governments worldwide, including Malaysia implemented targeted restricitons to protect the older adults.

Malaysia implemented one of the most prolonged and frequently reinstated lockdown strategies during the COVID-19 pandemic. The first nationwide Movement Control Order (MCO) began on 18 March 2020 and continued through four phases until 12 May 2020. This was followed by a Conditional MCO (CMCO) from 13 May to 9 June 2020**, a** Recovery MCO (RMCO) from 10 June 2020 to 31 March 2021, and repeated reintroductions of CMCO and Enhanced MCOs (EMCOs) in high-risk areas throughout 2020 and 2021. A strict Full MCO (FMCO) was again imposed nationwide from 1 June to 28 June 2021 [7]. Malaysia’s cultural values are strongly rooted in collectivism, where the needs of the group, particularly family and community, are prioritized over individual autonomy. Interdependence between generations is emphasized through traditions of filial piety, where adult children are expected to provide care and support for their aging parents, consistent with broader Asian cultural norms [8]. Strong family bonds, religious practices, and community support networks provided both material assistance and emotional comfort, supporting adaptation and fostering resilience in the face of prolonged isolation [9].

Only a handful of published studies conduct on the mental health effects of the pandemic have been on older adults, most of which were quantitative surveys, with even fewer studies considering qualitative evaluation among COVID-19 effects in older adults in low and middle-income countries (LMIC) in Southeast Asia [1,4,10,11]. Given Malaysia’s socio-cultural context and prolonged lockdown measures, it is important to examine how older adults navigated this crisis over time. A qualitative exploration allows for a deeper understanding of how they coped with the disruptions and perceived the long-term effects on their emotional and social well-being.

The objective of this study was to explore how older adults in Malaysia experienced, adapted and coped with social and emotional challenges posed by the COVID-19 pandemic. To obtain detailed descriptions of the personal experiences of older persons, identify coping mechanisms and adaptive strategies and examine the perceived long-term effects of the pandemic on the well-being of the individual’s mental health. The findings of this study would help identify potential strategies to avoid negative psychological consequences of future major disasters on the mental health of older persons.

## Materials and methodology

This study adopts a phenomenological ontology, which assumes that reality is subjectively experienced and meaning is constructed through lived experiences. This informs a constructivist epistemology, where knowledge is co-created through interpretation between researchers and participants. Accordingly, we employed in-depth interviews and thematic analysis to explore how older adults made sense of their experiences during the COVID-19 pandemic. The alignment of research elements, the phenomenological ontology (reality as lived experience) leads to a constructivist epistemology (knowledge as subjective and interpretive), which in turn justifies the use of qualitative, interpretive methods to capture participants’ perspectives authentically [12].

The study population was recruited using purposive sampling ensuring representation of individuals across different age, gender, marital status, living situation and other relevant sociodemographic characteristics [13]. Eligibility criteria required participants to be aged 60 years or older, ability to speak English or Bahasa Malaysia to understand the study participant information sheet and consent form and willing to participate voluntarily. However, all the interviews were conducted in English, with participants having good proficiency and comfort in expressing themselves in the language. The participants were recruited by listing the study in different WhatsApp groups postings shared within several community and interest-based groups that included older adults or individuals who had direct contact with them. A brief study invitation message was posted in these groups, including a contact number for interested individuals to call or message via WhatsApp. This method was chosen due to the widespread use of WhatsApp among older Malaysians and their family members for everyday communication. There were around 4 participants recruited through WhatsApp groups and from there snowball sampling, whereby initial participants referred friends or acquaintances. Once a participant or their family member expressed interest via WhatsApp or phone call, the researcher scheduled an initial phone conversation to explain the study objectives, procedures, and ethical considerations. No study website or email link was used; all communication was conducted via WhatsApp and telephone. On average, two to three phone calls were made before the interview: one to explain the study and schedule the appointment, a second if clarification was needed, and a final reminder sent via WhatsApp one day before the scheduled interview. A copy of the recruitment message has been included as a supplementary file (see Supplementary S2 File).

As researchers trained in mental health and gerontology, we acknowledge that our professional backgrounds, personal values, and cultural contexts inform our interest in the wellbeing of older adults, particularly during public health crises such as the COVID-19 pandemic. Our commitment to understanding and advocating for the emotional and social needs of older persons stems from both clinical experience and scholarly engagement with aging populations. Given the disproportionate burden older adults faced during the pandemic, we were particularly attuned to issues of loneliness, isolation, and resilience.

Individual, face to face, in depth, semi-structured interviews were conducted between May 2023 to March 2024, which was post-pandemic. The qualitative study is part of the the mixed method study, which utilized quantitative data from the TrAnsforminG cognitive frailty into Later-LifE Self-Sufficiency (AGELESS) study. It’s a collaborative effort between public and private universities, including, aiming to develop effective strategies to enhance the well-being of older adults. The AGELESS study combined three existing prospective studies which recruited individuals aged 55 years and over between 2013–2016, the Malaysian Elders Longitudinal Research (MELoR), Towards Useful Ageing (TUA) and Prevent Elder Abuse and Neglect Initiative (PEACE) [14,15]. While no participants were directly recruited from these cohorts for the current qualitative component, the development of the interview guide was informed by findings from the quantitative strand of the AGELESS study, which examined depression, anxiety, and stress among older adults during the COVID-19 pandemic. The qualitative phase aimed to further explore and contextualize these psychological outcomes through in-depth interviews. Participants were recruited independently, separate from the AGELESS cohort, to capture a broader range of lived experiences relevant to the study objectives. The interview guide was informed by the findings of quantitative analysis survey and developed with a panel of experts. The quantitative strand, derived from the AGELESS study, assessed psychological outcomes such as depression, anxiety, and stress among older adults during the COVID-19 pandemic. Building on those findings, the qualitative interview guide was designed to explore in greater depth the lived experiences and coping mechanisms of older adults.

The interview guide was informed by two complementary frameworks: the Social-Ecological Model of Resilience [16,17] and the Transactional Model of Stress and Coping [18]. The social-ecological lens guided exploration of how individual, relational, and contextual factors shaped participants’ resilience, while the stress and coping model helped structure questions around how participants appraised and managed pandemic-related stressors. Together, these frameworks supported a holistic exploration of coping and adaptation within the Malaysian cultural context.

These theoretical underpinnings ensured that the interview questions moved beyond surface-level descriptions and elicited nuanced accounts of psychological adaptation. While the interview guide did not directly replicate items from the AGELESS questionnaire, it was informed by the psychological constructs identified in the quantitative data. This helped ensure conceptual continuity across both strands of the study. The complete interview guide can be found in Supplementary File. The interviews were conducted in person by the researcher. Two pilot interviews were conducted prior to the main research interviews to test the interview protocol, refine the questions, and ensure the researcher was comfortable with the process before interviewing the actual participants. The time taken for interviews ranged from 16 to 60 minutes on average. The semi- structured interviews were recorded and stored in secure digital files. Study recruitment ended at the point of saturation where no new themes were identified during the analysis. A further three interviews were conducted to ensure data saturation. Data saturation was reached at 16 participants; however, three additional interviews were conducted to ensure thematic completeness and confirm consistency across responses.

### Data management and ethics

The participants, who had voluntarily agreed were given the letter of participation and consent form. The letter of participation included the purpose of study, procedures to be used in data collection and other necessary information related to the research. The written consent form included the permission for audio recording for the interview sessions. The researcher transcribed all the interviews and the information was kept confidential. The names or any personal identity was excluded from the transcript to maintain the confidentiality.

The audio recordings were kept securely. Hardcopy version of the data (e.g., questionnaires) were stored in locked cabinets in the university. Digital version of the data were stored in secure password protected computers. (all information and data will be stored on a secure, Health Insurance Portability and Accountability Act (HIPPA) complaint storage account). Only the researchers who had been briefed on the security measures of the project will have access to the data. The recorded interview audios, once transcribed would be kept for five years from the date of publication (thesis, journal article etc) before being destroyed or deleted completely [13]. This qualitative study obtained ethics approval from the University of Malaya Research Ethics Committee (UMREC), University Malaya, Kuala Lumpur, Malaysia (No. UM.TNC2/UMREC_2602)

### Data analysis

Data analysis was conducted using the Atlas.ti qualitative data analysis software [19]. All interviews were conducted in English; thus, no translation was necessary. The interviews were audio-recorded and initially transcribed verbatim by the researcher through careful listening. To ensure accuracy, the transcripts were cross-checked by re-listening to the recordings and re-importing the audio files into Atlas.ti, which provided an additional layer of verification before coding and analysis [19]. The transcripts were further triangulating with the field notes for consistency. To maintain anonymity, all transcripts were anonymised by removing names and any potentially identifiable information, and participants were assigned unique codes. The data collection and analysis procedures, including the use of Atlas.ti, were reviewed and approved by the university’s research ethics board.

The data analysis process was conducted using a phenomenological method, consisting of seven steps, offering a systematic approach to capture the essence of participants’ lived experiences [20].

Step 1 familiarisation of data: researchers read and re-read all the interviews transcribed verbatim to gain familiarity with the data. The transcript was anonymised and important notations or contextual information were inserted. Step 2 formulation of meaning: all the transcripts were read line by line, to capture the meaning emerging from the data. At the beginning of the data analysis the researcher transcribed two interviews and identified codes and discussed with the co-researchers to maintain the consistency of coding approach. In Step 3 meanings were formulated from the significant statements, a deductive coding approach, developing a coding framework based on established theories of positive psychology and coping [10]. This framework was then systematically applied to each transcript through line by line coding. Step 4 organizing formulated meanings into clusters of themes: the codes were chunked and then assessed to identify codes that were similar or redundant to reduce the number of codes. The researcher clustered the identified codes, grouping them into themes or subthemes that were common across all accounts. Throughout the process, regular meetings were held with co-researchers to discuss and debate and refine the codes and themes supporting creditability through collaborative analysis. Step 5 developing an exhaustive description: the phenomenological investigations were purposed to deepen the knowledge on the impact of COVID-19 on the mental health of older persons.. All data collected were charted by reorganising and summarising all the themes and their codes created in the previous steps in a chart for comparison of the data within and across interviews. Step 6 identifying the fundamental structure of the phenomenon: was embedded in the final stage of analysis by interpreting the data as a whole and synthesising key themes to capture the essence of participants’ experiences. Step 7 returning the findings to participants for validation: was not conducted due to logistical constraints. Nonetheless, rigour was maintained through triangulation, peer debriefing, and systematic analysis.

## Results

A total of 26 participants were invited for interviews. Four declined to participate, citing unwillingness to share their personal experiences. One participant did not attend on the scheduled interview day, and two others were unable to agree on a suitable time. Total of 19 participants agreed to be interviewed. Among the 19 participants who agreed to take part, 10 were women, with a mean age of 70 years (see Table 1). Two participants reported being diagnosed with obsessive compulsive disorder, anxiety, and depression before the pandemic. Additionally, fifteen participants reported medical diagnoses of hypertension, diabetes, arthritis, high blood pressure, stroke and heart disease. These health conditions were based on self-reported information shared during the interviews and were not clinically verified. Table 1 presents demographics of 19 participants, including gender, age, current living situation, education, employment status, occupation and marital status.

**Table 1:** Participant Demographics.

Participant ID	Gender	Age	Current Living Situation	Education	Employed/ Retired	Occupation	Single/ widowed
**P1**	Male	70	Family	Diploma	Employed	Business	Married
**P2**	Female	63	Spouse	Law	Employed	Lawyer	Married
**P3**	Female	81	Alone	Secondary school	Retired	--	Single
**P4**	Female	65	Family	Diploma	Employed	Insurance agent	Single
**P5**	Female	63	Family	Diploma	Employed	Receptionist at clinic	Single
**P6**	Female	79	Alone	Degree	Retired	--	Widowed
**P7**	Female	61	Alone	Degree	Retired	--	Single
**P8**	Female	78	Spouse	PhD	Employed	Professor	Married
**P9**	Male	76	Spouse	Diploma	Retired	--	Married
**P10**	Female	66	Family	Secondary school	Employed	Office job	Widowed
**P11**	Male	66	Family	Secondary school	Employed	Guard	Married
**P12**	Female	66	Family	Primary school	Employed	Office cleaner	Widowed
**P13**	Male	65	Family	Primary school	Employed	Selling vegetables	Married
**P14**	Female	64	Family	Primary school	Employed	Food stall	Widowed
**P15**	Male	81	Spouse	Degree	Retired	--	Married
**P16**	Male	70	Spouse	Degree	Retired	--	Married
**P17**	Male	76	Spouse	Masters	Retired	--	Married
**P18**	Male	61	Spouse	Degree	Employed	Engineer	Married
**P19**	Male	72	Spouse	Masters	Employed	Engineer	Married

Note: P = participants. “—” indicates not applicable. Occupation is listed only for participants who were employed at the time of interview.

Participants shared a diverse range of nuanced experiences from the onset of the COVID-19 pandemic. Through analysis, we identified four overarching themes, each encompassing multiple sub-themes that capture the complexity and depth of these experiences, these themes are presented in Table 2.

**Table 2:** Identified Themes and Sub-themes Related to COVID-19 Experiences.

Themes	Subthemes
Emotional and Psychological symptoms linked to infection control	• Fear of being infected and its consequences • Anxiety, worry and stress for self and other falling ill • Feeling depressed from social isolation
Feeling of loss from restrictions	• Loss of connection to people • Decline in physical activity • Loss of confidence and freedom • Loss due to death • Loss of purpose • Loss of financial security
Coping Strategies in Adversity	• Culturally grounded health practices • Grounding through mindfulness and nature • Adapting to connecting virtually • Shopping strategies and food accessibility • Coping through religious faiths
Information and communication	• Negative influence from news on social media • Connection through social media

### Theme 1: emotional and psychological symptoms linked to infection control

Most of the participants described experiencing significant emotional distress during the pandemic, manifested through fear, anxiety, worry, stress, and depression. The fear of infection, as well as the fear of spreading the virus to loved ones, was pervasive. This fear extended beyond the act of contracting the virus, encompassing uncertainty about its long-term consequences, which led to excessive precautionary measures, such as disinfecting groceries, avoiding close contact with others. The worry extended to the safety of family members, particularly the vulnerable, heightening stress levels. Prolonged isolation, disruptions to daily routines, and the inability to engage in previously meaningful activities, also contributed to feelings of hopelessness, low motivation and emotional fatigue. These emotional responses were not transient but became embedded in everyday life, underscoring the profound psychological impact of prolonged infection control measures.

#### Fear of being infected and its consequences.

Participants reported their fear of going out and being infected with the virus, which has been lingering throughout the pandemic and post-pandemic.

P7 mentioned….“there was also the fear of going out – the fear of going out and catching COVID and all sorts of things. Scared lah* *, not really anxiety because the auntie stand(sic) very close to me.“So even you buy toilet paper from Speedmart 99, you come back, I put it on my car and sun it for a while. “(June 2023)*

* a local expression used to punctuate speech, with no particular meaning

*Similarly P 17 mentioned…..“So after the COVID it really scared me, frightened me and then I start to think about all kind of consequences and yeah. Whether in future the health would (be) threatened and all kinds of thing.” (April 2024)*

These narratives illustrates, the psychological impact of COVID-19 extended into anticipatory fear about long-term health consequences. These narratives illustrate how the experience of illness reshaped participants’ outlook on health and ageing; not as isolated events but as disruptions that undermined their sense of safety and future stability.

Even after pandemic restrictions were relaxed, some participants continued to avoid crowded places, such as weddings or religious events. The persistence of these adaptations reflects a recalibrated sense of safety and social engagement.

*As P4 described…“So even though later on we are physical in (the) office I also refused to attend the meeting.. I say I go online in the office itself. They give me options ma (local expression)I refused to attend the physical meeting. Even when (the) boss call(s) me*

*I said,’no, no’ you (have) got five people inside. I stay outside.”(June 2023)*

The decision to remain physically distant, even when offered a choice between in-person or virtual attendance, reflects not just caution but a redefined relationship with space and safety, as shared environments are no longer perceived as inherently safe.

Through these lived accounts, it becomes evident that the fear of contagion was not a temporary response but a deeply embedded experience that redefined participants’ interactions with their social world.

#### Anxiety, worry and stress for self and others falling ill.

Participants described their concerns about them being within the high risk group to based on age alone. This concern extended to elderly parents with pre-existing health conditions and young grandchildren, prompting heightened vigilance and self-imposed restrictions. While they acknowledged their own vulnerability, many participants were more distressed about the possibility of becoming a vector of infection to loved ones.

*P1 clarified….. “Not afraid but anxious worried about grandchildren getting it-more worried about family members rather than myself.” (May 2023)*

*P4 described …..“I feel like I feel bit concerned about my mum therefore cannot go out*

*We (are) scared because she is elderly in her 80s so I am afraid we might spread the virus if you get it…. because at that time there is (sic) no vaccination*.” (*July 2023*)

The participants were worried about their family, other older people and their health and future. This reflects broader cultural norms of collectivism and filial obligation, particularly prevalent in many Asian societies, where protecting family, especially elders is both a moral duty and a source of identity, where care for others shaped emotional responses and everyday choices.

Feeling stressed and worried were common issues among the participants during the pandemic. They felt this was something new and didn’t have adequate information and guidance to deal with the uncertainty.

*P2 mentioned about unknown……“Especially the first lockdown it was something… we had to get used to… never happened before… totally… I didn’t like it. Every day you hear about the news, you know. What you hear on media and on TV, that really made you worried like what’s the world coming to.” (May 2023)*

*P7 reflected …..“And then at one point in KL, the lockdown was so bad that you cannot even go out again. I think middle of 2021, I realized that that is when I could feel the stress building up.*” *(June 2023)*

The constant state of uncertainty created a sense of confinement and heightened psychological tension, particularly during periods of strict enforcement or when performing essential tasks. This illustrates how repeated lockdowns created cumulative emotional fatigue among the participants. P2’s reference to the unknown highlights how different forms of uncertainty whether aleatory (stemming from the inherent unpredictability of the pandemic) or epistemic (arising from limited knowledge, evolving guidelines and conflicting information) shaped older adults’ risk perceptions. Such uncertainties intensified feelings of anxiety, stress and loss of control over daily life, underscoring how psychological strain was not just about isolation, but also about navigating the ambiguity of a rapidly changing crisis environment.

**P2* expressed…..“But when I felt unwell and I went to the doctors clinic that was very very stressful* *But I had to visit the doctor… I felt if anybody sits next to me… I will get uptight… If anybody coughs I will get uptight. Going to the doctor was very stressful.*“*(May 2023)*

What was once routine became a source of emotional distress. These accounts highlight how the pandemic reshaped everyday environments into spaces of fear, revealing the embodied nature of stress and its link to perceived vulnerability.

#### Social isolation and emotional well-being.

The participants who experienced illness during the pandemic, whether due to stroke or COVID-19, described profound emotional suffering, not simply from the physical illness but from the isolation that accompanied it.

*P9 reflected on the loneliness that accompanied his stroke recovery during the pandemic: ”In fact, what happened was during the,… during the COVID, of course, we have, we advise people not to come and many will stay away. But during my recovery from stroke, I didn’t want visitors because it was very depressing at that time. So depression was more due to my being isolated from a lot of activities that I was very interested in.” (Sept 2023). Here, the absence of visitors and withdrawal from valued activities created a sense of disconnection that compounded the physical vulnerability he was already experiencing. His narrative reveals how recovery, in isolation, became a deeply distressing and disorienting experience.*

Similarly, another participant spoke of the emotional aftermath of surviving COVID-19. Although his physical symptoms subsided, a persistent sense of unease and restlessness emerged, accompanied by disrupted sleep and a loss of emotional equilibrium:

*P17 stated “So even though I recover from it, but after recovering from it, very soon I went into depression. And then I had to rely on sleeping pills, to sleep, to take and I feel very restless. I mean about one month or two months after recovering, no after three (weeks) or one month after that, I feel (sic) really depressed.” (April 2024)*

The enforced isolation sometimes compounded by participants’ own precautionary behaviours led to a profound sense of withdrawal from the world, which impacted participants’ mental health severely illustrating that public health measures extended beyond physical threat of the COVID-19.

Together, these accounts reveal how illness during the pandemic extended beyond the physical body; deepening isolation, disturbing routines, and leaving emotional imprints that lingered long after recovery.

### Theme 2: Feeling of loss from restrictions

The COVID-19 restrictions brought about a profound sense of loss among older adults, affecting their social connectedness, autonomy, confidence, and sense of purpose. While physical needs were largely met, the disruption of everyday routines and interpersonal engagements led to experiences of emotional displacement and existential unease. This theme captures how participants internalised the cumulative impact of the restrictions, leading to shifts in identity, diminished self-worth, and feelings of psychological dislocation.

#### Loss of connections to people.

Participants described feelings of being disconnected with their loved ones, families and friends due to social distancing and travelling restrictions. Some participants had adult children who had migrated overseas and were unable to make their usual annual visits. Regular visits, once a meaningful anchor in their lives, were replaced by video calls, which felt emotionally inadequate.

*For example,* ***P18****, shared how virtual communication fell short of meeting emotional needs “No, once a year. But during COVID, we were on video call. Or maybe we do WhatsApp video… WhatsApp call. So that was also something which was painful. Not painful, it was we couldn’t meet her. We couldn’t see her. See the children.” (April 2024)*

His statement reflects a longing not merely for visual contact, but for physical closeness—holding, touching, and being in the presence of loved ones—essential elements of familial intimacy that were denied.

*Similarly P10 mentioned ……”The difficult part is sometimes not so difficult. What I mean is not so difficult. It’s just that you sometimes you have to… like you got somebody to talk to. Sometimes you go to the neighbour there, you chit chat. Maybe sometimes we have tea together. That’s all cut off.” (Jan 2024).*

This illustrates how even casual social rituals played a significant role in maintaining emotional well-being. Their disruption revealed how deeply embedded these micro-interactions were in participants’ sense of normalcy and belonging.

The loss was further intensified when major life milestones were missed.

*P13 expressed ……”So basically it’s more to... You missed your... I mean being a grandfather, you were not able to enjoy it. Yeah, yeah. That went through. You went through that part. It was a sad life.” (Jan 2024). His use of “sad life” points to a deeper existential pain and loss of intergenerationa connection.*

#### Decline in physical activity.

Participants described a disruption in their physical routines that previously provided structure, wellbeing, and social connection. The fear of infection, even in open spaces, led to self-imposed restrictions that altered their embodied habits and daily rhythms.

P2 mentioned…..*“Exercise was out. I used to exercise daily. But even going for morning walks also you felt apprehensive for some reason… Might bump into someone so I felt I will not do my usual routine. So the exercise daily part of my life changed a lot. I tried to exercise at home but myself I didn’t have the discipline.”(May 2023) P17 shared…“Almost two years grounded in the house. But of course I take my walk outside for exercise purpose and all this after a year of lockdown. Even doing exercise, playing with the group taiji was stopped for 2 years… Then I went for one eye checkup and three days later I was infected with COVID.” (April 2024)*

These narratives illustrates how physical activity, once a consistent and self-directed aspect of daily life, was disrupted by the pervasive sense of ambient threat, where even eventual re-engagement with physical activity came after long-term confinement. The suspension of group activities such as taiji underscores the dual loss—physical movement and communal interaction. Although walking outdoors is generally safe, the participant’s hesitation reflects the internalization of risk, routine medical visit further reinforces the sense that resuming normalcy carried lingering psychological risk.

These narratives demonstrate how the decline in physical activity was not just behavioural but existential; entangled with fear, loss of routine, and the collapse of embodied freedoms.

#### Loss of confidence and freedom.

COVID-19 restrictions led to a profound disruption in personal autonomy, especially in relation to mobility and self-reliance. The extended period of immobility caused some participants to experience a decline in self-confidence, particularly in tasks they had once performed routinely, such as driving. These losses were not only practical but deeply symbolic, touching on core aspects of identity, agency, and independence—values that often become even more meaningful in later life, when maintaining autonomy is closely tied to one’s dignity and self-worth.

*P6 reflected …….”That life has gone because of the pandemic.Because of the pandemic sort of it made your life too static you know. You were not allowed to move around you were not permitted to go out anywhere, you see. So you become like… on your own. And then after that I told you I became sick. Maybe if I hadn’t fallen sick… after the pandemic I still might have… might have the confidence to drive… to go distant places. I really lost my confidence.“(June 2023) P3 stated……”I use to drive here and there then because of the COVID, I (was) restricted. So is a loss of freedom……….Now I don’t drive…, feel not confident. I only recently about one month (ago), I sold my car.” (May 2023)*

Here, driving is not merely a functional activity—it represents a form of independence that promotes agency and fosters well-being The loss of confidence in performing this task marked a deeper shift in self-perception, from being capable and active to being dependent and uncertain. Her remark that “life has gone” suggests an existential sense of disruption, where the past self no longer feels accessible. In the context of older adulthood, selling a car carries more than logistical implications; it is not just a vehicle but a symbol of independence, and letting it go can signify a shift into greater reliance on others and a narrowing of one’s world, potentially impacting people’s self-worth and well-being.

*P3 further reflected……“It is not your own house. Only loss, you have you lost your own (house comfort). I lost my freedom in the way (dependent). In the sense, food wise and everything wise is provided for… buy coconut water… buy this buy that … everything buy…The thing is only your freedom.” (May 2023)*

Although her physical needs were met, the emotional experience of being cared for without control evoked a deep sense of loss. In older age, when maintaining control over decisions and routines becomes increasingly important for preserving identity and well-being, this feeling of disempowerment was especially difficult.

#### Loss due to death.

Loss of loved ones during the pandemic was described as emotionally devastating, particularly when public health restrictions disrupted traditional mourning rituals.

*P9 described his grief* ……“Then my brother also passed away during COVID, not due to COVID, you know, but because of the restrictions. Yes, I was able to pay my respects and come off (leave) immediately. They didn’t want to allow us to stay there too long, you know. So, even the funeral proper, they had it on Zoom. Yeah. because it happened during the COVID MCO* *, that was very traumatic because he was the youngest in our family.” (Sept 2023)*

* MCO = movement control order

His distress was not solely about death itself but the *incomplete grief*—being denied the culturally significant acts of communal grieving and physical presence. The phenomenological experience here includes an interrupted mourning process that left a lingering emotional void.

#### Loss of purpose.

Beyond physical and relational losses, several participants expressed a diminished sense of purpose. The loss of routines, social roles, and opportunities to contribute meaningfully to others left some individuals questioning the value and direction of their lives during the pandemic.

*P6* *shared how the abrupt halt of social and physical mobility disrupted her sense of direction: “That life has gone because of the pandemic… you become like… on your own” (June 2023)*

*P2 mentioned……..“I missed going to church. Maybe the social life with my church friends. Yes that part I missed. Because church was also locked down for at least one and half years or something like that.”(May 2023)*

Her repetition of “life has gone” indicates not just boredom but existential displacement—a shift from an active contributor to a passive observer. The closure of churches and community groups removed important roles for some participants. As P2 described missing her church involvement, it was not simply about socialising but the loss of embedded roles that gave her identity structure. Beyond this it also limited access to psychological comfort that could have helped participants master the stressful situation, meaning an additional source of support was missing to cope with adversity.

#### Loss of financial security.

For some participants, the pandemic disrupted not only their routines and relationships but also their financial stability, which posed a significant threat to their sense of self-reliance. The loss of income for some participants made accessing essential items more challenging, with some relying on community support to meet their needs. They expressed a deep sense of helplessness as their income dwindled, leading to significant lifestyle changes. Despite the passage of time, they reported that their income has yet to return to normal. Thus, the pandemic did only impact during the pandemic but had long lasting consequences.

***P13*** who ran a market stall, described how the six-month closure of the market severely affected his livelihood: “Market business… you can’t open, they closed the market. DBKL* *closed the market for six months… six months! So difficult for us to run (meet) daily expenses and all. After about two weeks later when the COVID (outbreak) closed down the market, we started off (again). Then we started doing home delivery. Then after that six months later they opened and come back to normal. But still after the COVID, business was not the same… like this.” (June 2024)*

His account reflects a disruption in economic routine, but also the loss of a social role. As a provider and worker, his identity was intertwined with daily business activity. The phrase “business was not the same” points to a lasting impact—not only financially, but in how the participant perceived their ability to recover and resume pre-pandemic life.

*P14* who ran a food stall business, reported pawning her jewellery in order to pay her employees’ salaries during the early phase of the pandemic: *“Very bad, because I got no (not) much savings. So I went and pawn all my jewels(jewellery). Oh, so financially. To pay all my workers. Pay workers’ mah, give them salaries. I have some problem… a lot.”(Sept 2024)*

Her account reveals the emotional toll of having to liquidate personal and symbolic assets, including items that may have held familial or cultural meaning. Jewellery, often a marker of security and tradition in many cultures, was sacrificed to meet obligations—underscoring her sense of responsibility in crisis but also personal loss.

### Theme 3: coping strategies in adversity

Despite the challenges posed by the pandemic, many individuals found support provided by family, friends, and community members. Practical help, such as setting up virtual communication tools or providing food, was invaluable in maintaining a sense of normalcy. Community and political support further reinforced the feeling of being cared for and not facing the crisis alone. Adapting to new routines became a significant aspect of coping during the pandemic to protect themselves from uncertainty and threat of COVID-19 infection. Participants described how they altered their routines, connected virtually, and relied on spiritual resources to maintain psychological stability. These protective strategies were not merely functional but deeply embedded in their lived experience of safety, belonging, and meaning-making.

#### Culturally grounded health practices.

Some participants relied on traditional knowledge and practices to cope and maintain their health. These routines were more than preventive strategies—they represented a source of control, self-efficacy, and identity in uncertain times.

*P1 described how he relied on natural remedies and home-prepared food as a preventive health strategy: I follow simple rule don’t eat out try not to eat out – eat home cooked food use good spices in Indian home- I take lot of garlic onion cloves pepper chilli pad I take all of this a lot and every night without fail one glass of hot water with turmeric- not affecting my health- I feel healthy- so keep on doing this follow- nature’s way of helping.” (May 2023)*

His detailed routine reflects not only a health practice but a resilient mindset rooted in cultural knowledge and personal conviction. By trusting in “nature’s way,” he enacted a form of control over his health, reaffirming his belief in the body’s ability to withstand external threats through natural means.

#### Grounding through mindfulness and nature.

Others coped by drawing inward—adapting mentally and emotionally through acceptance, mindfulness, and routines that created stability.

*P5 described she adapted to the new normal…..Then after I kind of got used to it I learn to accept it I learn to cope By making sure that I am always next to the window when I am in the room luckily my house has got all windows and then I can spend some time out in the porch so that helps. Of course meditation and that helps.Internet actually saved lot of people because you can get entertainment you can get so many things on internet.” (Jan 2024)*

This layered coping approach involved connection to nature, mindfulness, and purposeful use of digital tools. Her response demonstrated psychological flexibility and acceptance—key attributes of behavioural adaptation.

#### Adapting to connect virtually.

While physical distancing disrupted familial roles and routines, some participants reframed their experiences by using digital platforms to stay connected with loved ones.

*P15 expressed …….“Normally we would go and visit the other son in Australia… But during lockdown, we didn’t do anything… Well, thanks to technology… we can still use WhatsApp and also Zoom.”(April 2024)*

*Similarly P6 was grateful…And very often my friends visit me and they come drive here I go with them and buy whatever I need so I felt I don’t really need the car so let him have it (June 2023)*

**P6 who previously attended in-person religious classes, described how the shift to online sessions gave her renewed purpose. ”I used to go for my religious classes in the evening.* *When the MCO came, I couldn’t go for those classes so it left a void in the schedule and later on I am not sure whether the same year or the next year they found Zoom (a video conferencing platform) and they started conducting Zoom classes. I was very happy. Someone came and set up everything so I just follow I just follow… so the password is there, just press (touch screen) on the password and then ok, press and I get into in the class”.(June 2023)**

This adaptation illustrates emotional regulation through gratitude and relational continuity, where maintaining emotional connections despite physical separation helped buffer feelings of isolation and loss.

#### Shopping strategies and food accessibility.

Participants expressed a strong fear of being in crowds due to the risk of contracting the virus. This led them to make significant changes to their daily routines, including minimizing outings and making do with available resources for extended periods.

*P19 described how he and his household adjusted the timing of shopping trips to reduce risk: “So during the COVID, take extra precautions and then also … we choose a time choose a*

*time you know. During the time when there are less people we think when there are less people so it create less problems.” (April 2024)*

“we” suggests that decisions were made together, showing that safety was a shared concern. Choosing specific times to go out reflects a careful effort to avoid risk while still keeping some control over daily life

*P7 a practising Buddhist, vividly described how her spiritual beliefs supported her mental health “I limit myself … I limit myself. Each time if I go out I make sure I have to get everything and it has to last me for a week or two you know That means I have to stretch my need to go out as much as possible. Of course the fridge become stocked up lah.” (June 2023)*

Her efforts to limit outings show how carefully she managed her safety. Keeping the fridge well-stocked wasn’t just practical—it gave her a sense of security and readiness during an uncertain time.

#### Coping through religious faiths.

Several participants drew strength from their religious and spiritual practices, which provided emotional regulation, structure, and meaning during periods of isolation. Faith was described as both anchor and lens, shaping how participants interpreted and responded to confinement. Through prayer, meditation, or community connection, participants established new norms within constrained environments.

*P5* *who felt confined by physical restrictions, found comfort in her home’s architectural openness and her spiritual routines:“Then after I kind of got used to it I learn to accept it I learn to cope By making sure that I am always next to the window when I am in the room luckily my house has got all windows (windows on all sides)… and then I can spend some time out in the porch so that helps. Of course meditation and that helps.” (Jan 2024)*

Her experience highlights a phenomenological shift from confinement to containment—where her home transforms from a prison to a sanctuary. The window and porch become symbolic thresholds, linking her inner life to the outside world, while meditation offers a way to sustain psychological spaciousness.

*P7 mentioned….”I tell some of my Buddhist friends if I had no Dharma I would be cuckoo already. Because the Dharma is like an anchor for us now. Even though we were all locked in quarantine but we still communicated through Zoom, through WhatsApp.” (June 2023)*

Her statement underscores the protective and interpretive power of faith, framing Dharma not only as spiritual guidance but as a psychological stabiliser. The use of “anchor” reflects how her belief system grounded her sense of reality during upheaval. Even in physical isolation, virtual communication sustained her community and preserved her sense of shared meaning.

These adaptive behaviours—ranging from altered routines to virtual engagement and spiritual practice—illustrate how older adults actively negotiated safety, connection, and purpose. Rather than passive recipients of care or regulation, participants demonstrated resilience and resourcefulness, adapting their lifeworlds to the realities of the pandemic while maintaining continuity in what mattered most to them.

### Theme 4: information and communication

The role of media and social communication during the pandemic was multifaceted. On one hand, access to news and digital platforms provided essential updates and enabled social connectedness. On the other hand, participants described feeling overwhelmed by the constant stream of messages—particularly on social media—which often heightened fear and uncertainty. This theme explores how older adults experienced the emotional weight of risk communication, as well as the comfort of staying connected through digital means.

#### Negative influence of news on social media.

Many participants expressed concern over the news they encountered on social media. They felt overwhelmed by the constant flow of information on platforms like WhatsApp, which fuelled their anxiety and fear about the spread of the virus.

*P2* *described how daily exposure to media reports amplified her fears about the state of the world: “Every day you hear about the news you know. What you hear on media and on tv that really made you worried like what’s the world coming to.”(May 2023)*

*P7* shared how social media, rather than calming her, increased her anxiety symptoms *“Ya la (a local expression), you know somebody say something and then you Google. Then you know follow That time… not afraid… until more social media makes me more afraid.” (June 2023)*

These narrative shows that the constant flow of grim or alarming news contributed to a broader sense of existential uncertainty. The phrase “what’s the world coming to” indicates not just concern for personal safety but a disruption in her worldview—one in which the future felt unstable and unpredictable. This reaction aligns with the literature on risk communication, where intense media coverage can unintentionally escalate public fear and undermine psychological resilience [21,22]. The shift from “not afraid” to becoming afraid illustrates the cumulative psychological impact of ambiguous or exaggerated digital messaging, particularly when individuals are trying to make sense of rapidly changing circumstances.

#### Connection through social media.

While Social media could amplify anxiety, participants also acknowledged its important role in fostering connection and maintaining routines. Digital communication became a lifeline to emotional support allowing them to stay connected with family and friends and attend religious virtual gatherings.

*P5* *expressed appreciation for how technology helped her stay in touch with colleagues from her religious community: “Of course because of internet, because of telephone because of WhatsApp Also because I was work from home at that time I was also connected with my Buddhist society colleague.” (Jan 2024)*

##### Similarly, P15 described how digital tools allowed for safe yet meaningful interaction:

*“I think as long as you try to stay indoors, you know, minimal outing, you can still communicate with friends, loved ones, you know, and nowadays with the WhatsApp and video”.(April 2024)*

These narratives shows that experience reveals the dual role of technology—not only as a communication tool but as a means of preserving identity and social roles during a time of disruption. Staying connected with her religious group allowed to maintain a sense of belonging and continue spiritual engagement, reinforcing structure and meaning in day-to-day life. WhatsApp and video calls became substitutes for face-to-face interactions, allowing older adults to sustain their relationships despite isolation.

Together, these narratives reveal how the COVID-19 pandemic profoundly reshaped the psychological, social, and cultural lives of older adults. Emotional distress linked to infection control, feelings of loss, and the lack of confidence were not isolated experiences but interrelated disruptions that resonated across daily routines, relationships, and self-perceptions. Digital technologies, mass media, and social communication further amplified this duality exacerbating anxiety and fear in some instances, while offering vital pathways for connection and meaning in others. The risk communication is important to reduce the spread of virus, it is equally important to consider how such messages are perceived, especially when always older adults are framed as a high risk group [4].

## Discussion

This study aimed to explore the social and emotional impact of the COVID-19 pandemic on older adults. The findings revealed a shared experience of uncertainty and emotional strain, shaped by fear of infection, social isolation, and disruptions in their daily routines. Participants reported various forms of loss, including loss of social connections, mobility, independence, and, in some cases, purpose. Despite these challenges, many demonstrated adaptability through personal coping strategies and support from family and community. These systems of support, along with individual resourcefulness, underscored the ability to adapt and overcome adversities, reflecting aspects of resilience [16] Understanding these experiences is crucial for informing future public health strategies and providing targeted support to older adults in our setting and other LMICs with similar economic and cultural status in future major disasters. While global pandemics are once in a century events, major disasters in terms of major floods, landslides, heat waves, poor air quality, and epidemics do occur regularly within Malaysia [23], and globally older persons in LMICs are also particularly vulnerable during other disasters including drought, forest fires, earthquakes, war and conflict, and economic crisis. Particularly those with limited mobility, chronic health conditions or reduced access to social and medical support.

The pandemic and protective measures created tensions and ambivalence across individual and social levels [24]. This is further supported by a qualitative study in Italy in 2021, which highlighted common coping strategies used to reduce exposure to COVID-19. These included measures such as wearing masks, baking mail, disinfecting groceries, washing hands frequently, and discussing cleaning practices obsessively. These actions were often undertaken to protect oneself, family members, and others [25]. The fear of infection triggered irrational behaviours, highlighting growing concerns about anxiety and stress, which ultimately may impact the psychological well-being of older adults.

The sense of loss from both official and self-imposed restrictions led to disruptions of regular activities. Physical activity is important for older adults, especially to maintain their level of independence, mental health, and well-being. A longitudinal study in the UK, conducted between 2015 and 2020, found that reduced physical activity during the COVID-19 pandemic was associated with increased symptoms of depression and anxiety among adults over the age of 50 [26]. Social distancing may make people feel safer, it can also increase their feelings of isolation, stress and frustration and cause difficulties in many life situations [27] While many expressed hope for a return to normalcy, the persistence of new cases, even as the pandemic transitioned to an endemic phase, continued to fuel fear and uncertainty. The expressions of diminished purpose align with established conceptualizations of well-being. Ryff’s eudaimonic model of psychological well-being identifies purpose in life as a core dimension [28], and Keyes’ model of social well-being emphasizes social contribution as essential to feeling useful and connected to society [29]. The disruption of roles and routines during the pandemic challenged older adults’ sense of direction and self-worth, highlighting how loss of purpose can deeply affect emotional well-being.The restriction on movement heightened the fear of going out, further diminishing confidence due to the loss of freedom. As a result, participants stopped driving, struggling to regain their pre-pandemic routines.

Social isolation resulted in feelings of restriction, particularly regarding social and family interactions, participation in leisure activities, and shifts in work relationships, which left older adults dependent on others. A qualitative study conducted across Brazil, Portugal, Chile, and Spain in 2022 further highlighted how older adults experienced disruptions to their daily routines and increased dependence on family members, reinforcing the emotional and practical impact of prolonged isolation [6]. The participants received significant support from family, friends, neighbours, and the community which included assistance with purchasing daily necessities and ordering food. Additionally, participants from lower socio-economic backgrounds expressed gratitude for the aid provided by the community and political organizations, which supplied food and essential items.. The Socioemotional Selectivity Theory suggests that older adults prioritize emotionally meaningful relationships, and thus, social distancing measures that limited face-to-face interactions may have heightened feelings of loneliness among this group [30].

Traditionally, it has been considered the norm and cultural practice within Malaysian society for adult children to take care of their older parents. However, it is not only the living arrangement, but it is the combined effects of living arrangements and strong social support networks which influence the mental health status of older Malaysians [8]. Family members remained the most significant source of support for the participants. A scoping review across African countries in 2023 [31] and a qualitative study in Wuhan, China in 2021 [32] both underscored the central role of family in providing emotional and psychological support. Older adults viewed their families as essential for psychological support, as communication with them helped alleviate negative emotions.

Social distancing is a preventive strategy, but it can lead to social isolation and feelings of loneliness. During lockdown, individuals often isolated themselves to comply with restrictions and protect vulnerable populations. The cancellation and postponement of social events, as well as the avoidance of close interactions with others, have been associated with heightened feelings of loneliness, aligning with the Social Theory of Loneliness [33]. A study conducted in China during early stages of COVID-19 reported the social network of the older person often shrinks because of retirement, leading to a sense of social isolation has gradually increased [34]. Similarly, a qualitative study in Indonesia conducted in 2022 found that the loss of annual visits from distant adult children, disruption in their daily routines had contributed to the feeling of loneliness and social disconnection [5], which could further exacerbate isolation linked to retirement. Participants demonstrated the ability to adapt through adaptation to the new norm, modifying daily routines and embracing virtual platforms for social and religious activities. The shift to online communication and gatherings provided a sense of continuity, though the lack of in-person interaction was felt deeply. Participants also displayed resilience by finding ways to cope with restrictions, including meditation, maintaining a connection with nature, and fostering spiritual practices. These adaptive strategies reflect that adaptation is one aspect of fostering resilience in the face of adversity and highlight the potential of targeted interventions to promote mental well-being [35].

Older adults, particularly those living alone, relied on the digital platforms to stay in touch with family and engage in social interactions. These online activities reduced their stress levels and enhanced their interpersonal communication. While media and technology played a dual role during the pandemic, they were both a source of connection and a contributor to anxiety. This dual role of media underscores the importance of effective risk communication. Risk communication during public health emergencies must be timely, accurate, and credible to reduce uncertainty, promote appropriate protective behaviours, and prevent unnecessary fear [36]. Clear and targeted messaging is especially vital for vulnerable populations like older adults. A cross-sectional study conducted in China during the COVID-19 pandemic found that older adults experienced elevated levels of loneliness, depression, anxiety, and post-traumatic stress disorder, underscoring the importance of accessible and culturally appropriate health communication [37].

The older adults, particularly those who were self-employed or running small businesses suffered economic loss and food insecurity. Home-based older adults workers faced more challenges during the COVID-19 pandemic [38]. The lockdowns cancelled many social events resulting in lack of customers or passengers, affecting the businesses like food catering, motor taxi driving or construction work. These contributed to feeling of sadness and stress, due to loss of income and being unable to provide for their families [5]. Malaysian government allowed Employment Provident Fund (EPF) withdrawals to assist those affected financially due to COVID-19 pandemic, which helped working age adults but also reduced their retirement saving [39]. In Malaysia the retirement age is 60 years, by then part of their retirement savings have been withdrawn for family, medical, housing loan or other purposes. Malaysian retirees run out of EPF savings in less than 5 years after retirement [40]. Few retired Malaysians aged over 65 years will, therefore, have benefitted from the government mitigation policy of EPF withdrawals. Instead, many older adults are financially dependent on their adult children, who may have needed to withdraw their EPF savings during the pandemic to help sustain their older parents, if they had experienced loss of income, This could deplete their own long term retirement savings, potentially compromising their financial security in future [38].

Interviews were conducted after the pandemic, allowing for a comprehensive understanding of the long-term effects and post-pandemic impacts on older adults. This timing provided valuable insights into how individuals processed their experiences, adapted over time, and coped with the challenges, offering a deeper perspective on the enduring consequences of the pandemic. Participants included older adults who were retired as well as those actively involved in some form of work, which appeared to result in varying perspectives and experiences regarding the impact of lockdowns on their work and financial situations. Participants included both retired older adults and those still engaged in informal or part-time work, leading to differing perspectives on how lockdowns affected their financial and occupational roles.

The COVID-19 pandemic has changed the way people live, some of the problems older adults posed were fear of being infected, anxiety, stress, social isolation and loneliness. These factors suggest, that the mental health of older adults should be monitored post-pandemic period to support them in coping with social and psychological impact [41,42], while also resonating with regional studies in Asia that highlight culturally specific concerns, such as filial support and intergenerational dependency [43]. In the Malaysian context, many older adults relied on adult children for financial support, and in some cases, children were compelled to withdraw their EPF savings during the pandemic, potentially compromising their own financial future. This underscores the need for tailored interventions that address not only the psychological but also the socio-economic impact of the pandemic.

Overall, the findings contribute to the growing international literature by offering a culturally grounded, post-pandemic perspective from an upper-middle-income Asian country. In the absence of extensive English-language studies from Southeast Asia, this study adds depth to our understanding of how older adults in collectivist societies navigate crisis, highlighting the enduring need for mental health monitoring and financial support mechanisms in the post-pandemic period.

## Conclusion

In this study, the older adults shared their feelings of loneliness, fear, anxiety, and social isolation during the COVID-19 pandemic. Although adapting to the new norm was a challenge, older adults displayed resilience through coping mechanisms such as seeking family support, utilizing technology, and drawing on faith, hope, to manage stress. Longer term effects including loss of confidence, financial loss and health deficits have occurred. The resilience of older adults may be a useful resource in future major disasters, challenging current strategies to shield and provide. Future research should also determine strategies to mitigate the lingering negative psychological, physical and financial effects of the COVID-19 pandemic.

## Supporting information

S1 File Questionnaire/Interview guide. (DOCX)

S2 File WhatsApp Invitation Message for Research Interview. (DOCX)
