# Supplementary material for: Exploring the social and emotional impact of COVID-19 on older residents of the Greater Klang Valley, Malaysia: A qualitative study
Source: PLoS One. 2025 Oct 9;20(10):e0332610. doi: 10.1371/journal.pone.0332610 (PMC12510558; doi:10.1371/journal.pone.0332610)
Supplement: S1 File — (DOCX) [file pone.0332610.s001.docx]

**S1. Questionnaire/Interview guide**

**Opening statement**

Hello, My name is Shobha. I am from University Malaya. I am conducting a research for phd on the impact of COVID-19 on mental health of older person(60 and above). I am here to talk to you about your experience during the COVID-19 Pandemic.

Let me give you an outline of what’s going to happen. I am going to ask you a series of questions to understand things from your perspective. There are no right or wrong answers to any of the questions. We can talk a lot quicker than we can type, would it be OK with you for us to record this session for our note taking? We have prepared a consent form for you to review and sign. If at any point you want to take a break or stop the interview please just let us know and we can work around it.

Any questions before we begin?

Let’s get started!

**Semi-structured Questionnaire for Qualitative Research**

1. Tell me about your mental health during COVID-19.

2. What were your primary emotions at that time? ( using Primary emotion chart)

3. How did you manage the everyday task during COVID-19? (e.g. grocery

shopping, visiting doctors or pharmacy) How were these different from what you

did before?

4. Did you or anybody living with you get COVID-19? How was the experience?

Did you get hospitalised or had to isolate yourself? Were you at anytime

restricted doing things ?

5. How did you cope during the pandemic ? What helped you at that time? What

could have helped at that time?

6. Is there anything else about your experience with the current COVID-19

pandemic that you would like to share that we haven't talked about

already?
