# Supplementary material for: Exploring the social and emotional impact of COVID-19 on older residents of the Greater Klang Valley, Malaysia: A qualitative study
Source: PLoS One. 2025 Oct 9;20(10):e0332610. doi: 10.1371/journal.pone.0332610 (PMC12510558; doi:10.1371/journal.pone.0332610)
Supplement: S2 File — (DOCX) [file pone.0332610.s002.docx]

**S2: WhatsApp Invitation Message for Research Interview**

We are conducting a research study to understand the experiences of older adults during the COVID-19 pandemic. If you are aged 60 years and above, we invite you to participate in a voluntary face to face interview (approximately 45–60 minutes) to share your thoughts and experiences.

Your participation will help us better understand how the pandemic has affected the lives and well-being of older people. All information shared will be kept confidential and used only for research purposes.

If you are interested or would like to know more, please WhatsApp or call [researcher’s phone number].

Thank you for your time and consideration.
